# Chemical composition of the essential oils of *Rhodiola rosea* L. of three different origins

**DOI:** 10.4103/0973-1296.71782

**Published:** 2010

**Authors:** Ljuba Evstatieva, Milka Todorova, Daniela Antonova, Jordanka Staneva

**Affiliations:** *Institute of Botany, Bulgarian Academy of Sciences, Acad. G. Bonchev Str., Bl. 23, 1113 Sofia, Bulgaria*; 2 *Institute of Organic Chemistry with Centre of Phytochemistry, Bulgarian Academy of Sciences Acad. G. Bonchev Str., Bl.9, Sofia, Bulgaria*

**Keywords:** Essential oil, geraniol, myrtenol, phenethylalcohol, *Rhodiola rosea*

## Abstract

*Rhodiola rosea* L. (Crassulaceae), or “rose root” is a perennial herbaceous plant, distributed in the Northern Hemisphere. Pharmacological studies have shown that *R. rosea* exhibits different biological activities – antioxidant, antidepressant, anticancer, etc. The aim of this study was to compare the chemical composition of essential oils from rhizomes of three commercial samples of *R. rosea* originated from Bulgaria (sample 1), China (sample 2) and India (sample 3). The oils were analyzed by GC and GC-MS. Thus, the main volatile component in the Bulgaria and Chinese *R. rosea* was geraniol, followed by myrthenol in sample 1 or octanol in sample 2. Phenethylalcohol was a principal constituent in the Indian oil. Myrtenol and octanol were in significant amounts too. Aliphatic hydrocarbons were characteristic of the latter sample. It is notable that cinnamyl alcohol, which was present in large concentration in Bulgarian sample, was not detected in the other two samples. The obtained results showed considerable differences in the composition of the studied three origins of *R. rosea*.

## INTRODUCTION

*Rhodiola rosea* (Crassulaceae), commonly known as “rose roots”, or “golden roots,” is a perennial herbaceous plant. It is widely spread in the mountain regions of Central and Northern Europe as well as Asia and North America. Rose roots have been used in traditional and modern medicine for the treatment of different diseases.[1] In recent years, root extracts are applied as ingredients of food additives and other commercial pharmaceutical preparations offered all over the world. A great deal of focus has been put on this species and its medical properties with regard to memory and learning, immune response, organ function, cancer therapy, etc.[2–6] Phytochemical investigation of rose roots has been directed mainly on salidroside, rosin, rosavin, and rosarin.[7,8] Other important constituents of *R. rosea* are flavonoids, tannins, gallic acid and its esters, and essential oils.[9] The most detailed results have been reported on essential oil of *R. rosea* from Norway,[10] Finland[11] and Mongolia.[9]

In the present study three origins of *R. rosea* are compared in terms of their essential oil composition.

## MATERIAL AND METHODS

### Plant material

*R. rosea* commercial rhizomes imported to Bulgaria from India and China, as well as rhizomes from plants cultivated in Bulgaria, were used for this study.

### Preparation of essential oil

Ground rhizomes of each sample were subjected to microdistillation/extraction in Likens-Nickerson apparatus, using diethyl ether as a solvent. The latter was removed and the yield was presented in % w/w.

### Gas chromatography

Gas chromatography (GC) analysis was carried out on an HP-5890 instrument fitted with HP-5 MS capillary column (30 m × 0.25 mm), 0.25 µm film thickness; carrier gas was nitrogen. The injector and detector temperature was 260°C, column temperature was programmed from 50 to 230°C at a rate of 4°C/min and for 10 min at 230°C. Automatic integration of FID peak areas gave the amount of the components in percentage.

### Gas chromatography-mass spectrometry

GC-mass spectrometry (GC-MS) analysis was performed on an HP 6890 instrument equipped with MS detector, which operated in EI mode. All the conditions were as described for GC analysis but the carrier gas was helium. The oil components were identified by comparison of their mass spectra and retention indices with those published[12] or presented in Willey library.

## RESULTS AND DISCUSSION

Dry roots of *R. rosea* (samples 1, 2, and 3) were found to contain 0.21, 0.10, and 0.25% (w/w %), respectively, of pail yellow oil. GC analysis resulted in identification of 25 [Table 1] individual compounds, at concentration more than 0.20% at least in one of the studied oils. The identified components represent more than 85% of the total oils. Phenethylalcohol (56.22%) was the most abundant in the Indian oil. Myrtenol (10.56%) and 1-octanol (5.30%) were present in significant amounts too. Aliphatic hydrocarbons like nonadecane, heneicosane, docosane tricosane, tetracosane, and pentacosane (14.57%), characteristic of this oil were in lower amount in the oils from Bulgaria and China. Geraniol was the principal volatile component of Chinese (56.97%) and Bulgarian (48.79%) *R. rosea*, followed by myrtenol (28.05%) in Bulgarian or 1-octanol (12.21%) in Chinese sample. It is notable that cinnamyl alcohol that was present in a large concentration in Bulgarian sample was not detected in the other two samples. Geraniol and phenethylalcohol were identified as main rose like odor compounds. They were the characteristic components of essential oil from *Rosa* species.

**Table 1:** Chemical composition of *R. rosea* essential oils

RI*	Components	Bulgaria (sample 1)	China (sample 2)	India (sample 3)
978	1-Octen-3-ol		0.80	
992	6-Methyl-5- hepten-2-ol		0.44	
1001	Octanal		0.23	
1070	1-Octanol	0.33	12.21	5.30
1074	*cis*-Linalool oxide		2.16	
1098	Linalool	0.74	2.95	
1110	Phenethylal-cohol	0.65	4.19	56.22
1146	Isopulegol	0.22		
1189	α-Terpineol	0.34	0.80	0.64
1194	Myrtenol	28.05	0.85	10.56
1228	Citronellol		1.01	1.86
1255	Geraniol	48.79	56.97	3.69
1270	Geranial		1.63	
1283	*trans*-Anethol			0.97
1287	*p*-Cymen-7-ol		0.77	
1295	Perilla alcohol		0.56	
1300	Cinnamyl alcohol (E)	9.97		
1340	4-Vinyl-2-OMe phenol		0.64	0.98
1383	Geranyl acetate		0.76	
19.00	Nonadecane		0.16	2.51
2100	Heneicosane	1.82	1.16	3.57
2200	Docosane			0.51
2300	Tricosane		0.40	5.84
2400	Tetracosane	0.20		0.32
2500	Pentacosane			1.82
	Total	91.11	88.69	94.79

* RI - relative to C_8_–C_22_ *n*-alkanes on HP-5 column

This investigation shows that the main essential oil components in *R. rosea* from commercial plant material cultivated in Bulgaria and from natural habitats are identical,[13] i.e., its chemical composition is genetically determined. Different chemical composition of oils prepared from commercial plant sources of Bulgarian, Indian and Chinese origin could be due to the fact that they were from different species of genus *Rhodiola*, or were different chemotypes of *R. rosea*. On the other hand, Rhichard *et al*. reported that “Very often products called “*Rhodiola* spp., Tibetan *Rhodiola* or Indian *Rhodiola*” may incorrectly imply equivalence with *Rhodiola rosea* extract.”[3] Thus, the commercial material from Rhodiola has to be explored botanically and phytochemically.
